# Cdc42 controls secretory granules morphology in rodent salivary glands in vivo

**DOI:** 10.1080/19420889.2020.1724605

**Published:** 2020-02-11

**Authors:** Akiko Shitara, Christopher K. E. Bleck, Roberto Weigert

**Affiliations:** a Laboratory of Cellular and Molecular Biology, Center for Cancer Research, National Cancer Institute, National Institutes of Health, Bethesda, MD, USA; b Intracellular Membrane Trafficking Section, National Institute of Dental and Craniofacial Research, National Institutes of Health, Bethesda, MD, USA; c Department of Pharmacology, Asahi University School of Dentistry, Mizuho, Japan; d Electron Microscopy Core Facility, National Heart Lung and Blood Institute, National Institutes of Health, Bethesda, MD, USA

**Keywords:** Exocytosis, Cdc42, secretory granules, intravital microscopy, salivary glands

## Abstract

We previously reported that the small GTPase Cdc42 negatively regulates endocytosis in the salivary gland of live mice. By using intravital subcellular microscopy, we showed that depletion of Cdc42 causes the mis-sorting of plasma membrane components into intracellular vesicles, ultimately leading to the impairment of the homeostasis of the apical plasma membrane. In this study, we report that, besides, Cdc42 depletion alters the ultrastructure of large secretory granules analyzed by transmission electron microscopy. We found that lack of Cdc42 increases the number of granules per cell and alters their structure. Specifically, granules are smaller, less circular and exhibit heterogeneous electron densities in their lumen. Our findings suggest a novel role for Cdc42 in controlling granule biogenesis and/or maturation.

## Introduction

Specialized secretory cells contain large exocytic vesicles, termed secretory granules, which transport a large variety of proteins. Their size differs depending on the cell type, with exocrine organs having among the largest granules (often referred to as zymogen granules) [1]. Granules are generated from the *trans-Golgi network* (TGN) and undergo a maturation process after their release into the cytoplasm [2]. Based on studies in neuroendocrine cells and Drosophila, it has been proposed that granule maturation may occur by two different but non-mutually exclusive pathways, and specifically via 1) homotypic fusion of newly formed secretory granules [3–5]; and 2) vesicular-mediated retrieval of selected components from the immature granules [6–8]. Due to the post-explant de-differentiation of exocrine cells [9], secretory granules are usually lost in primary cultured cells. Therefore, the mechanisms of their maturation remain still largely unclear, at least for mammalian exocrine models.

Cdc42 is a small GTPase that activates various effectors regulating selected processes which include: membrane trafficking, the establishment of polarity, cytoskeleton reorganization, proliferation, and migration [10]. In a recent study, we showed that Cdc42 maintains and develops the apical plasma membrane in the salivary gland of live mice by negatively regulating endocytosis at the plasma membrane [11]. Here we extended our observations on the effect of Cdc42 depletion on regulated exocytosis. By using transmission electron microscopy, we found that lack of Cdc42 in the salivary gland acini of adult mice significantly increases the number of secretory granules, reduces their size and alters their content, thus supporting the idea that Cdc42 may regulate the biogenesis and/or maturation of the secretory granules, possibly after sorting and release from the TGN.

## Materials and methods

### Animals and procedures

All experiments were approved by the National Institute of Dental and Craniofacial Research (NIDCR, National Institute of Health, Bethesda, MD, USA), National Cancer Institute (NCI, National Institute of Health, Bethesda, MD, USA), and Asahi University (Gifu, Japan) Animal Care and Use Committee. Rosa^mT/mG^ mice were purchased from Jackson Laboratory (Bar Harbor, ME, USA). Cdc42 floxed mice were a generous gift of Dr. Yi Zheng (Cincinnati Children’s Hospital Medical Center, Ohio). Aquaporin5^Cre^ mice were a generous gift of Dr. Zea Borok (University of Southern California, California, CA, USA). All the mice (males and females) used in this study weighed 20–40 grams. Mice were anesthetized by an intraperitoneal injection of a mixture of ketamine (100 mg/kg) and xylazine (20 mg/kg).

### Microscope and imaging parameters

Fluorescence images were taken by a point-scanning IX81 inverted confocal microscope equipped with a Fluoview 1000 scanning head (Olympus America Inc., USA). All images were acquired using a Plan Apo 60x N.A. 1.42 oil immersion objective (Olympus America Inc., USA). Fluorophores were imaged using the appropriate lasers as required by their excitation spectra (laser excitation 405 nm, 488 nm, 561 nm or 633 nm). The optimal focal plane for imaging the acinar cells was set at ~15 µm below the surface of the gland. Z- stacks were acquired with a step size of 0.50–1.00 µm. And the pinhole was optimally set to 0.9 µm.

### Intravital subcellular microscopy

As previously described [11,12], submandibular glands of anesthetized mice were exposed and connective tissue was separated from the glands without injuring the parenchyma. Exposed glands were gently pulled out taking care of avoiding tissue damage. Mice were placed on the heated pad (37–38 °C) to maintain the body temperature. The externalized submandibular glands were accommodated on a coverslip mounted on the stage above the objective and constantly moistened with a carbomer-940-based gel (Snowdrift Farm, Tucson, AZ, USA) covered with lens cleaning tissue paper.

### Transmission electron microscopy

The gland tissue was excised and fixed for 90 min in 2% glutaraldehyde, 2% formaldehyde (Electron Microscopy Sciences, Hatfield PA, USA), in HEPES buffer (pH 7.2), post-fixed in aqueous 1% osmium tetroxide, block stained with 1% uranyl acetate, dehydrated in graded ethanol solutions, and embedded in Embed-812 (Electron Microscopy Sciences, Hatfield, PA, USA). Thin sections were stained with uranyl acetate, and lead citrate then examined on a JEM-1200EX (JEOL USA) transmission electron microscope (accelerating voltage 80 keV) equipped with an AMT 6 megapixel digital camera (Advanced Microscopy Techniques Corp, USA).

### Image analysis and quantitation

Analysis of number, size, and circularity of intracellular vesicles was performed with a transmission electron microscope. Vesicles were traced manually and analyzed using Fiji (NIH, Bethesda, MD, USA). Data analysis was done in Prism (Graphpad, San Diego, CA, USA). The volume rendering was performed with Imaris (Bitplane, Belfast, United Kingdom) using the isosurface tool. The structure of intercellular canaliculi and cell shape was traced according to the fluorescence of phalloidin and mTomato/mGFP, respectively.

## Results

To analyze the role of Cdc42 in the maintenance of the apical plasma membrane in the salivary gland epithelium, we used mice floxed for Cdc42 that were crossed with the Cre Recombinase (Cre) reporter mouse strain Rosa^mT/mG^ (Cdc42^fl/fl – mTmG^) [13,14]. In the absence of Cre, mice express a membrane-targeted peptide fused with the fluorescent protein tandem-Tomato, which upon Cre expression is replaced by a GFP-tagged membrane-targeted peptide [15]. To deplete Cdc42, we expressed Cre via adenoviral particles injected into the salivary ducts of adult mice [16]. Using intravital subcellular microscopy we investigated the defects in the dynamics of membrane trafficking induced by the lack of Cdc42. However, due to the low efficiency of Cre transduction in the salivary acinar cells (less than 1% of the epithelial cells) [16], we could not use electron microscopy to investigate the ultrastructure of the endomembrane system and in particular of the secretory granules. To overcome this issue, here we use another established strategy based on crossing the Cdc42^fl/fl – mTmG^ mice with a strain that expresses Cre under the control of the salivary gland-specific promoter aquaporin 5 (aquaporin5 ^Cre^) [17]. This approach ensures the ablation of Cdc42 in all the acinar cells since embryonic day 15 [11].

**Figure 1.**
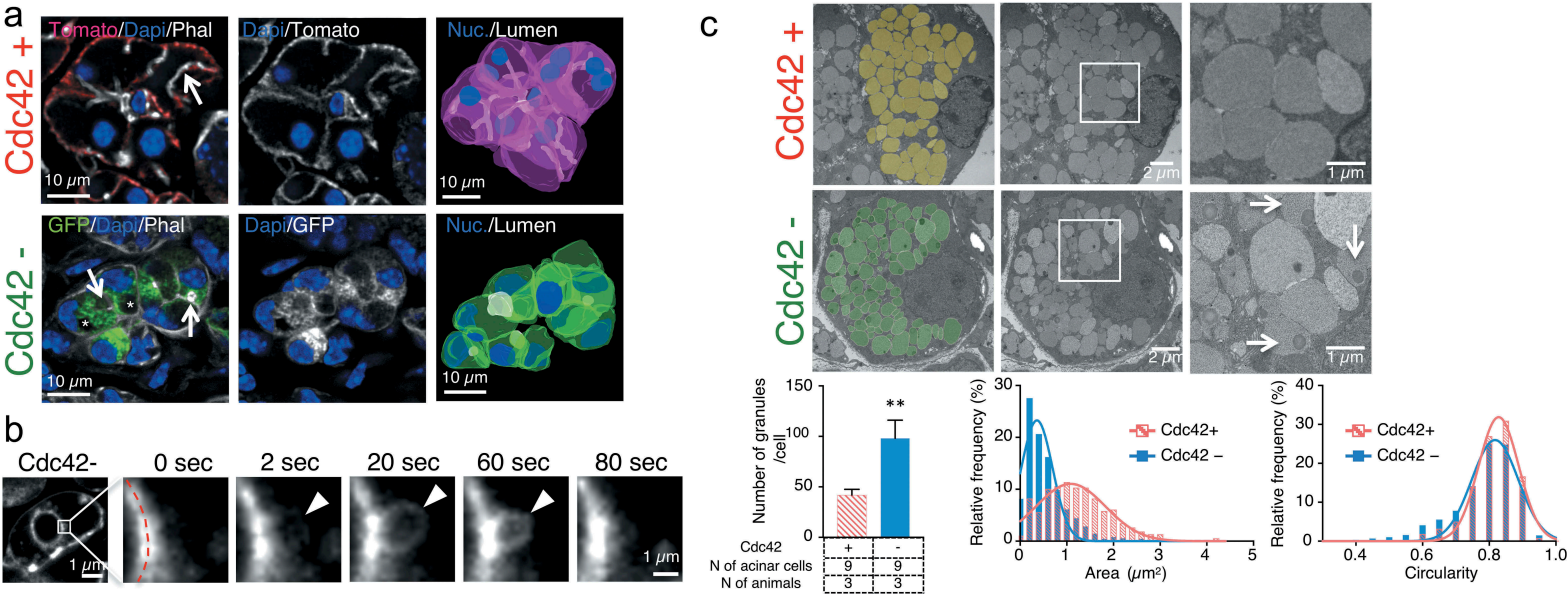
Cdc42 regulates the morphology of secretory granules in salivary glands. (a): Submandibular gland from mT/mG mice (upper panels) or aquaporin5^Cre^-Cdc42^fl/fl-mT/mG^ (lower panels) mice were fixed, processed, and labeled with Alexa 647-Phalloidin (white) and Dapi (blue). In Cdc42+ cells, the mTomato marker (red or white) is restricted to the plasma membrane, whereas in Cdc42− cells, mGFP (green or white) was observed also in intracellular vesicles and vacuoles (asterisk). Arrow indicates the lumen of the acinar canaliculi. Right panels feature the volume rendering of representative acini (Nuclei highlighted in blue and canaliculi highlighted in white; see also Supplemental Movies S1). (b): Regulated exocytosis was analyzed by intravital subcellular microscopy. Aquaporin5^Cre^-Cdc42^fl/fl-mT/mG^ mice were injected subcutaneously with 0.02 mg/kg isoproterenol, and integration of secretory granules into apical membrane was visualized by mGFP fluorescence (white), as previously described [14]. The time-lapse series of the integration of the secretory granules is shown in the right panels. Arrowheads and red dotted-lines show membrane fused secretory granules and apical membrane, respectively. Time 0 represents the point at which the limiting membranes of the secretory granules were detected. The integration of the secretory granules is shown in Movie S1. (c): Transmission electron microscopy of the submandibular salivary glands excised from Cdc42+ (top panels) and Cdc42− mice (middle and bottom panels). Individual acinar cells are represented in the left (secretory granules highlighted in colors) and middle panels. The high mag of the secretory granules is shown in the right panels. Arrows show secretory granule with heterogenous lumens. Graphs show the quantitation of the number of secretory granules per cell (left), area (middle), and circularity (right) of the secretory granules, respectively. Values were normalized by the total number of granules (378 granules for Cdc42+ cells in three animals; 882 granules for Cdc42- cells in three animals).

In control mice, the membrane-targeted Tomato was almost exclusively localized at the plasma membrane in the acinar cells of the salivary glands (Figure 1a, upper panel). In Cdc42-depleted cells, the membrane-targeted GFP was accumulated in intracellular vesicles and large vacuoles and not just at the plasma membrane (Figure 1a, Supplemental Movies S1). Intravital subcellular microscopy, revealed that regulated exocytosis was not impaired, as we previously observed using the adenovirus-based model (Figure 1b, Supplemental Movies S2) [11].

By using transmission electron microscopy, we observed that Cdc42-depleted cells exhibited twice the number of secretory granules than in control cells (Cdc42+ granules/per cell: 42 ± 3.2; and Cdc42- granule/cells: 98 ± 10.4. Average ± S.E.M; ** p < 0.01: unpaired Student’s t-test). Moreover, the granules were smaller in size (Cdc42+: 1.26 ± 0.04 µm^2^; and Cdc42-: 0.55 ± 0.02 µm^2^. Average ± SD; p < 0.01: unpaired Student’s t-test) and although their average circularity was slightly different (Cdc42+: 0.81 ± 0.01; and Cdc42-: 0.78 ± 0.01. Average ± SD; p < 0.01: unpaired Student’s t-test), a small fraction exhibited an irregular shape (Figure1c). Finally, the secretory granules in Cdc42-depleted cells exhibited heterogenous electron-dense areas in the lumen, which were not detected in control acinar cells (Figure1c, arrow).

## Discussion

Using transmission electron microscopy, here we show that Cdc42 regulates secretory granule morphology in the submandibular gland of adult mice. Specifically, the deletion of Cdc42 increased the number of secretory granules and altered their size and structure. Similar results were previously shown in PC12 upon overexpression of Cdc42 [15], thus suggesting that the Cdc42-mediated regulation of the secretory granules is tissue-specific, although a direct comparison should be performed within the same tissue. Since Cdc42-depleted acinar cells maintained regulated exocytosis [11], we argue that the smaller size and the heterogeneity of the lumen of the secretory granules are consistent with a defect in either their biogenesis from the TGN or their maturation. One possibility is that Cdc42 negatively regulates the biogenesis of the secretory granules, with a lack of Cdc42 resulting in the acceleration of the budding and/or fission of secretory vesicles, thus affecting the sorting process. As a result, smaller secretory granules with missorted cargoes (e.g. plasma membrane component) would form and be released from the TGN. Alternatively, Cdc42 could regulate the maturation process after the release of the secretory granules from the TGN. Secretory granules generally mature in a multi-step process which involves: homotypic fusion of small immature granules, acidification of the granular lumen, processing and aggregation of cargo proteins, and removal of excess membranes and proteins via either a clathrin-mediated or a clathrin-independent mechanism [2]. Although homotypic fusion has never been detected in the exocrine gland, the heterogenous electron-dense areas observed in the secretory granules are consistent with the formation of condensing vacuoles, which are a form of immature secretory granules [18,19]. Finally, Cdc42 could inhibit the removal of excess membranes during granule maturation, for example through clathrin-coated vesicles [8], which also affects granule size [20]. This process is topologically and functionally similar to endosomal recycling where Cdc42 has been shown to play a crucial role [21,22].

In conclusion, this study shows a novel role of Cdc42 in regulating secretory granule morphology *in vivo* that has not previously recognized. Additional work is required to further characterize this process and to unravel its precise molecular mechanisms.
